# An updated review of idiopathic osteosclerosis of the jaws

**DOI:** 10.21142/2523-2754-0803-2020-050

**Published:** 2021-03-11

**Authors:** Paul Marcelo Ñahuincopa López, Luis Miguel Romero Rodríguez

**Affiliations:** 1 School of Dentistry, Universidad Cientifica del Sur, Lima, Peru. marelo53@hotmail.com, luisromero.dksa@gmail.com Universidad Científica del Sur School of Dentistry Universidad Cientifica del Sur Lima Peru marelo53@hotmail.com luisromero.dksa@gmail.com

**Keywords:** Osteoesclerosis, panoramic radiography, cone beam computed tomography, mandible, osteoesclerosis, radiografía panorámica, tomografía computarizada de haz cónico, mandíbula

## Abstract

**Objective:**

: The aim of this study was to update the concepts of the diagnosis of idiopathic osteosclerosis (IO) of the jaws by digital panoramic radiographs and cone beam tomography and describe the impact of this disease on oral and general health.

**Methods:**

A search of the main databases of dental medical research was carried out using the search terms “osteosclerosis, panoramic radiography, cone beam computed tomography, jaws”. Articles without language restriction until September 30, 2020 were identified. The prevalence and clinical and radiographic characteristics of IO of the jaws were examined in 2D and 3D imaging studies, as well as the interaction during treatments in the various dental specialties.

**Results:**

We analyzed the current situation regarding the diagnosis of IO, with an update of the diagnostic criteria used to accurately identify IO in the latest generation imaging studies, as well determine its possible interactions in oral an general health.

**Conclusions:**

It is important to have a clear differential diagnosis of IO and be able to distinguish different radiopacities in the maxilla. Accurate reporting and monitoring of the morphometric characteristics are necessary taking into account the impact the presence of IO of the jaws has on future dental treatments.

## RESUMEN

**Objetivo:**

El propósito de esta investigación fue actualizar las consideraciones para el diagnóstico de la osteoesclerosis idiopática en radiografías panorámicas digitales y tomografías de haz cónico, a fin de determinar sus implicancias en la salud oral y general.

**Métodos:**

Se realizó las pesquisas en las principales bases de datos de investigación médica estomatológica, utilizando las palabras “osteoesclerosis”, “radiografía panorámica”, “tomografía computarizada de haz cónico” y “mandíbula”. Se identificaron artículos sin restricción de idioma, desde las primeras publicaciones hasta el 30 de septiembre del 2020. Se examinaron la prevalencia, las características clínicas y radiográficas en estudios imagenológicos de dos y tres dimensiones, así como su interacción durante los tratamientos realizados en las diversas especialidades estomatológicas.

**Resultados:**

La información obtenida nos permitió analizar la situación actual con respecto al diagnóstico de la OI y actualizar los criterios diagnósticos para una identificación certera de la OI en los estudios imagenológicos de última generación, así como sus posibles interacciones en la salud oral y general.

**Conclusiones:**

Es importante tener un criterio diagnóstico diferencial claro al distinguir las diferentes radiopacidades como la OI, que se pueden presentar en los maxilares, mediante un registro preciso de sus características morfométricas y seguimiento en el tiempo, teniendo en cuenta su existencia y sus implicancias en los tratamientos dentales a futuro.

## INTRODUCTION

In oral and maxillofacial radiology imaging studies of multifactorial etiology, it is important for dentists to be able to recognize incidental radiological findings ^1^^-^^3^, with differential diagnostic criteria. Auxiliary studies with digital panoramic radiography (DPR) ^(2, 3)^ and cone beam computed tomography (CBCT) ^(4, 5)^ allow visualization of anatomical structures and asymptomatic findings of clinical relevance, such as idiopathic osteosclerosis (IO) ^(2, 6-8)^.

**Figure 1:**
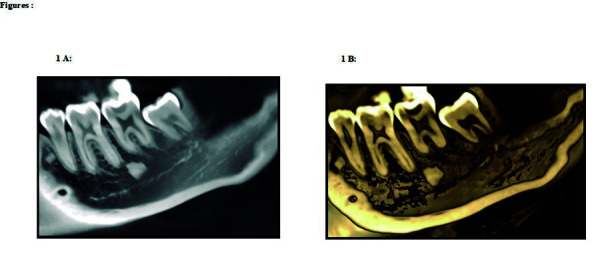
A, 3D reconstruction showing the imaging characteristics of IO visualized as dense, calcified osteosclerotic foci with a homogeneous background. B, Panoramic image of the IO adjacent to the dental roots

IO is considered an incidental finding ^(1, 3)^, being radiopaque, and with an estimated prevalence of 1.96% ^9^^)^ to 26.9% ^10^. The characteristics of IO are a dense, calcified osteosclerotic focus with a homogeneous background, absence of medullary spaces ^1^, without a corticalized halo, variable in size, and single or multiple in number ^11^. While in most cases IO appears adjacent to the dental roots (Figure 1A , 1B), it can also appear in edentulous areas without expansion of bone cortices ^1^ and is stationary ^1^. It is more frequent in the mandible (95%) ^12^, generally around or superimposed on the inferior dental nerve (IDN) canal ^13^; however, it can also occur in hip and long bones ^14^^,^^15^.

The prevalence of IO includes the premolar ^7^^,^^16^ and mandibular molar regions ^7^^,^^11^^,^^12^^,^^17^^,^^18^, and is more frequent between the second and fourth decade of life ^7^^,^^15^^-^^17^, mainly in women ^2^^,^^4^^,^^5^^,^^7^^,^^15^^-^^17^^,^^19^. It is also more common in African, Japanese, Chinese and Indochinese populations ^20^, which is important to take into account in multi-ethnic countries.

Some researchers consider IO as an anatomical bone variant which occurs during development, with no treatment and only follow-up being performed ^6^^,^^7^^,^^11^^,^^12^^,^^21^. Non recognition of the presence of IO and the absence of criteria for the differential diagnosis of this disease makes diagnosis and treatment difficult. In addition, IO can disrupt tooth eruption leading to alterations during dental positioning in the maxillae until impaction ^1^^,^^14^^,^^19^^,^^22^, or complications during movement in orthodontics ^14^^,^^22^, causing external root resorption associated with first permanent molars in 10 - 12% of patients ^20^. Regarding the impact and interrelationship with treatments of IO in different specialties, extension and overlapping of IO in the mandibular canal can lead to the need to modify pre-surgical planning of oral rehabilitation implants ^7^ in order to avoid increases in temperature during drilling, denaturation of bone proteins and irreversible osseointegration ^8^^,^^23^. In addition, there are inconveniences in relation to anesthesia of the IDN, with a high recurrence rate being reported in the literature, despite surgical removal ^8^ ^22^. Ignorance of its radiological characteristics of OI and their clinical relevance may lead us to unnecessary biopsies ^13^.

In the literature, IO is also known as enostosis, osteosclerosis focus, periapical osteopetrosis or bone scar ^8^^,^^22^^,^^24^^-^^28^. There is currently no literature guiding the differential diagnosis of IO ^1^^,^^3^.

The aim of this study was to update the concepts of the diagnosis of IO of the jaws by DPR and CBCT and describe the impact of this disease on oral and general health.

## ARTICLE SELECTION

A search was carried out in Medline stomatological medical research databases via PubMed, Scopus, EBSCO, Science Direct, SciELO and LILACS, using the words “idiopathic osteosclerosis, panoramic radiography, cone beam computed tomography, dentistry, oral radiology, orthodontics, oral implantology”. Sixty-four articles were identified, 36 of which met the selection criteria, being analyzed without language restriction, from the first publications until September 30, 2020. The prevalence, clinical and 2D and 3D radiographic characteristics, their interaction during the treatments carried out by different stomatological specialties. Finally, descriptive observational study designs, and studies on prevalence and case reports were included due to the lack of other types of studies.

## UPDATED CRITERIA FOR THE DIAGNOSIS OF IO OF THE JAWS

DPR is used as an auxiliary study to complement the clinical diagnosis of IO which is a common incidental finding to bone condensation ^1^^-^^3^^,^^6^^-^^8^. IO is frequently reported as a single focus ^2^ in the premolar and molar area in the mandible ^2^^,^^6^^,^^7^^,^^11^^-^^13^^,^^17^^,^^18^^,^^31^ in 95% of the cases between the second and fourth ^2^^,^^7^^,^^17^, and sometimes in the fifth decade of life ^7^^,^^12^. It is more prevalent in women ^2^^,^^4^^,^^5^^,^^7^^,^^19^^,^^17^^,^^19^. and is found in the incisor, canine, premolar and molar regions in 3.7%, 3.7%, 44.4%, and 48.2%, respectively, of the cases ^7^. The most frequent localization is in the dental root in the apical area ^2^^,^^7^^,^^32^, separated from the root ^14^^,^^17^ or between roots ^2^. The prevalence ranges between 1.96% to 11.8% ^2^^,^^3^^,^^7^^,^^9^^,^^12^^,^^14^^-^^21^^,^^25^^,^^32^^-^^34^.

**Figure 2:**
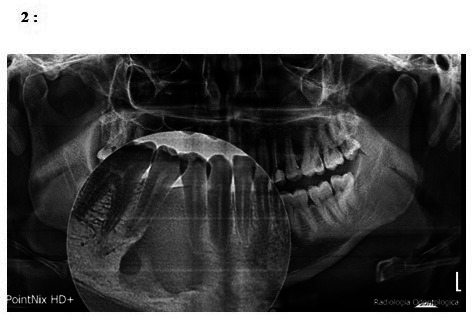
IO of considerable size that includes the mean reference measurements from 33.9 ± 20.1 mm2, with a height of 7.7 ± 3.1 mm and a width of 6.6 ± 3.1 mm

The mean measurements of references with respect to the area of IO are 33.9 ± 20.1 mm^2^, with a height of 7.7 ± 3.1 mm, width 6.6 ± 3.1 mm, and distance from IO to the mandibular midline of 26.6 ± 10.7 mm and 9.7 ± 3.7 mm at the mandibular border ^7^ (Figure 2).

In the DPR study IO is shown as avidity, minimal distortion, density and adequate contrast, without the presence of radiographic artifacts or evidence of tumors or trauma. The common characteristics of IO shown by DPR include a radiopaque appearance with a homogeneous background, rounded shape, with regular or no borders, that do not have a radiolucent halo, no mimicking bone trabeculae no thickening of the lamina dura, single or multiple foci ^2^, within the vicinity of dental roots and in edentulous areas in some cases ^2^.

## UPDATED CRITERIA FOR THE DIAGNOSIS OF OI BY CBCT

**Figure 3:**
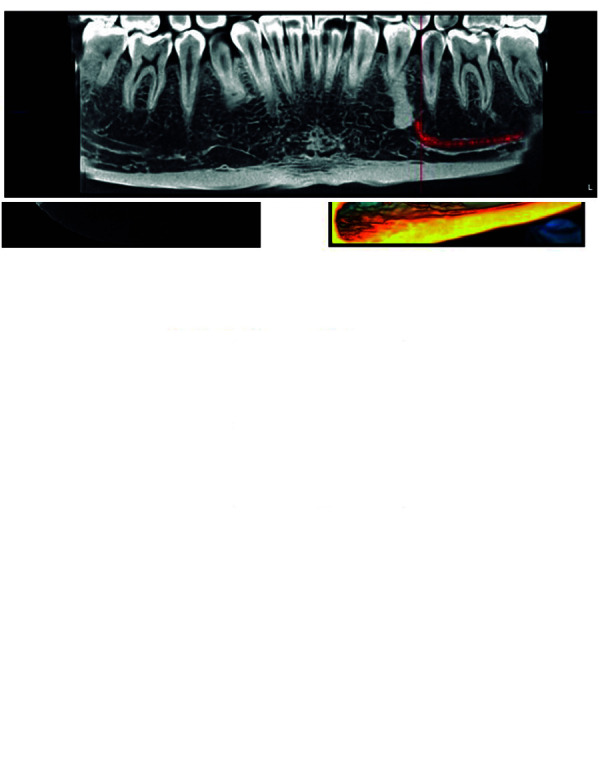
A: Sagittal section of the IO in cone-beam computed tomography. B: 3D reconstruction of the IO. C: Panoramic slice of the IO showing proximity to the premolar and the inferior dental nerve canal. D: Axial View of the IO.

CBCT provides a differential confirmatory study due to the possibility of obtaining different slice proportions with volumetric reconstructions of great 3D avidity at a real 1:1 scale, completely eliminating the overlapping of anatomical structures, and using a lower radiation dose with respect to conventional tomography. Uniform, round or oval hyperdense images can be very clearly located in the middle of the bone trabeculae, without surrounding hypodense areas surrounded by bone with normal radiographic characteristics. The CBCT study also determines relationships with dental roots and other important anatomical structures such as the IDN ^8^^,^^22^^,^^24^^,^^25^ (Figure 3A, 3B, 3C, 3D).

IO most frequently appears in the third decade of life ^26^, with a prevalence of between 16.7% ^26^ and 26.9% ^10^ and is more common in the lower (82.6% ^10^ to 95.7% ^26^), than in the upper jaw (17.4%) ^10^, and in the premolar region (29.9%) ^26^ molar region (52.7%) ^26^ separated from dental roots ^10^. The mean size ranges 1.5 mm to 15.6 mm ^26^^,^^35^.

The characteristics of IO are that of a radiopaque round mass ^27^ or hyperdense area ^28^, with a uniform background, surrounding a bone trabecular lesion without structural alteration ^27^.

## UPDATED CRITERIA FOR THE DIFFERENTIAL DIAGNOSIS OF IO WITH DPR AND CBCT

For the differential diagnosis of IO DPR is a preliminary examination and the characteristics of CBCT allow definitive diagnosis ^13^ taking into account the following clinical and radiographic criteria: the presence of circumscribed radiopaque images associated with large restorations, fixed or removable partial prosthetic abutment teeth, endodontic treatments associated with chronic processes with reactive focus such as condensing osteitis or chronic focus sclerosing osteomyelitis, mixed bone condensations with diffuse borders and different degrees of radiopacity (radiolucent-radiopaque), with the appearance of fibro-bone lesions or odontomas, cement-bone dysplasia characterized by mixed areas. Hypercementosis is shown as a hyperdense image which alters root shape preserving the lamina dura and periodontal ligament space, while cementoblastoma causes root resorption, presenting a radiopaque image with a radiolucent halo. A history of extracted teeth with the presence of root is identified by the presence of lamina dura and the space of the periodontal ligament, torus, or single or multiple bone exostoses that radiographically present as well-defined radiopacities, salivary stones, calcified lymph nodes, calcified stylohyoid ligament, calcifications of nutritional canals, foreign bodies, impacted teeth, osteocondensation with evidence of cortical expansion, patients with evident intraosseous pathology and / or craniofacial malformations in people over 40 years of age ^1^^-^^8^^,^^11^^,^^13^^-^^15^^,^^17^^-^^19^^,^^20^^-^^25^^,^^29^^,^^30^^,^^32^.

## IMPLICATIONS OF THE PRESENCE OF IO IN THE JAWS

An incidental radiographic finding of IO is considered by many researchers as an anatomical variant of normal bone during its maturation and development process. It is generally asymptomatic, does not require treatment, and only follow-up is necessary ^1^^-^^3^^,^^6^^,^^7^^,^^11^^,^^21^.

**Figure 4:**
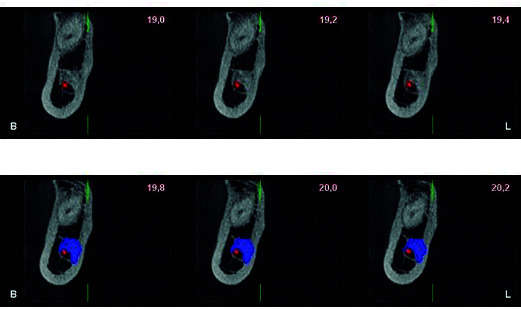
A, B: Sagittal view of the IO showing its proximity to the inferior dental nerve canal.

The presence of IO is clinically associated with obstruction or deviation of the dental germ leading to alterations in the spatial position of teeth in the maxillary arches and even impaction ^1^^,^^14^^,^^20^^,^^19^^,^^22^. In orthodontic treatment, this can cause difficulties during alignment movement, leveling and closure of spaces ^14^^,^^22^^,^^30^, causing external root resorption associated with the first permanent molars ^20^. Overlapping of IO to the mandibular canal can be asymptomatic ^35^, and is associated with persistent idiopathic orofacial pain or neuralgic pain due to compression of the trigeminal nerve, causing discomfort in the inferior dental nerve canal, and producing neuropathies ^(24, 25)^. (Figure 4A, 4B) There may also be difficulties in trunk nerve block ^(8, 22)^, suggesting the need for modifying pre-surgical planning during oral implant rehabilitation ^7^. A specific protocol of saline irrigation at 5 °C may be required to avoid an increase in temperature at the time of drilling denatured bone matrix proteins, negatively affecting the primary stability of the implant and osseointegration processes ^(8, 23)^. The incidence of recurrence is high after surgical removal of IO, requiring the need for resection with safe margins ^28^. It should be taken into account that differential diagnosis would avoid the need for biopsy ^13^. A statistically significant correlation was also demonstrated with the presence of IO in patients with chronic liver failure due to the presence of hepatic osteodystrophy ^36^. These patients can present idiopathic dental pain in healthy teeth without restorations. Indeed, in patients with hepatocellular carcinoma IO led to external reabsorption of the mesial root of the lower first molar ^27^, intermittent paresthesia of the mandibular body, and half of the tongue, lip and lower incisor area due to invasion of IO in the dental canal ^2^.

## DISCUSSION

This review provides updated diagnostic criteria to detect IO and interpret interactions with related entities. The etiology of IO is idiopathic, and the disease is generally asymptomatic. However, in the last decade there have been reports involving a complex diagnostic process. The prevalence of IO can be determined using DPR ^3^^,^^29^^,^^23^, while taking into account radiographic artifacts due to overlapping images that can be difficult to interpret when IO is present in the anterior area ^22^. The use of CBCT has demonstrated the presence of regional block of the mandibular nerve by proximity to IO ^8^^,^^22^, partial paresthesia of the lower lip or orofacial pain with characteristics of neuropathic pain ^8^^,^^10^^,^^22^^,^^24^^-^^28^^,^^35^ due to compression in branches of the incisive nerve ^28^ and by compression of the IDN by IO, causing neuropathies such as neuropraxia, axonotmesis, neurotmesis ^24^^,^^25^^,^^35^.

Clinicians must become familiar with the detection and identification of bone condensation with the use of differential diagnostic criteria. IO is generally associated with healthy teeth without previous treatment and may interact with future treatments. In orthodontics and maxillary orthopedics, it is important to report cases with impactions, difficulties in closing spaces and deviations in teeth position ^1^^,^^14^^,^^19^^,^^20^^,^^22^^,^^30^. In endodontics, idiopathic odontalgias are related to external resorption of the third apical root of the tooth in the proximity of IO ^20^. In cases of IO in implantology, it is necessary to apply a specific drilling protocol with irrigation of an isotonic solution at 5 °C ^8^^,^^23^. In oral and maxillofacial surgery, accurate differential diagnosis can avoid the need for biopsy ^13^, and it should be taken into account that a high incidence of recurrence has been reported following the resection of IO ^28^. Apart from oral medicine, IO has also been described in long bones and hip bones ^14^^,^^15^. Moreover, one study described a statistically significant correlation of IO in patients with chronic liver failure ^36^. The management of IO requires an auxiliary DPR study. In addition, prior to the analysis, interpretation and identification of IO, CBCT should be performed to evaluate the characteristics using 2D and 3D images providing data related to the presence of single or multiple foci, disease extension, the relationship with the dental roots, and proximity to other anatomical structures to be taken into account during follow-up.

This review highlights the importance of the incidental detection of IO, requiring the need for follow-up of this entity due to its interaction with other diagnostic entities.

## CONCLUSIONS

Timely detection of IO is important due to the possible serious impact this disease may have on oral health. It should be taken into account that many odontalgias of idiopathic nature can lead to erroneous therapeutic procedures such as unnecessary root canal treatments and extractions of healthy teeth, with persistence of the initial idiopathic pain irreversibly affecting the quality of life of the patient.

The identification of IO can be achieved by DPR which provides a detailed description of the localization of IO radiographic follow-up over time. However, in cases in which the clinical diagnosis is difficult and idiopathic pain of unknown origin persists CBCT should be performed. 
